# Genome-Wide Identification and Expression Profiles of Late Embryogenesis-Abundant (LEA) Genes during Grain Maturation in Wheat (*Triticum aestivum* L.)

**DOI:** 10.3390/genes10090696

**Published:** 2019-09-10

**Authors:** Datong Liu, Jing Sun, Dongmei Zhu, Guofeng Lyu, Chunmei Zhang, Jian Liu, Hui Wang, Xiao Zhang, Derong Gao

**Affiliations:** 1 Key Laboratory of Wheat Biology and Genetic Improvement for Low & Middle Yangtze Valley, Ministry of Agriculture/Lixiahe Agricultural Institute of Jiangsu Province, Yangzhou 225007, China; 2 Yangzhou University, Yangzhou 225009, China

**Keywords:** late embryogenesis-abundant gene, wheat (*Triticum aestivum* L.), grain maturation, genome-wide identification

## Abstract

Late embryogenesis-abundant (LEA) genes play important roles in plant growth and development, especially the cellular dehydration tolerance during seed maturation. In order to comprehensively understand the roles of LEA family members in wheat, we carried out a series of analyses based on the latest genome sequence of the bread wheat Chinese Spring. 121 *Triticum aestivum* L. *LEA* (*TaLEA*) genes, classified as 8 groups, were identified and characterized. *TaLEA* genes are distributed in all chromosomes, most of them with a low number of introns (≤3). Expression profiles showed that most *TaLEA* genes expressed specifically in grains. By qRT-PCR analysis, we confirmed that 12 genes among them showed high expression levels during late stage grain maturation in two spring wheat cultivars, Yangmai16 and Yangmai15. For most genes, the peak of expression appeared earlier in Yangmai16. Statistical analysis indicated that expression level of 8 genes in Yangmai 16 were significantly higher than Yangmai 15 at 25 days after anthesis. Taken together, our results provide more knowledge for future functional analysis and potential utilization of *TaLEA* genes in wheat breeding.

## 1. Introduction

The dehydration process of embryos during grain maturation is the terminal event of seed development, correlating with the acquisition of desiccation tolerance and with the specific induction of highly abundant hydrophilic proteins described as Late Embryogenesis Abundant (LEA) [1,2]. This process indicates the “switch” of seed transformation from development to germination, acting as an adaptability strategy of angiosperm to ensure the survival of seeds during storage or the environmental stress of long-term evolution [3,4]. Seeds are classified as orthodox and recalcitrant based on their storage behavior and desiccation tolerance in the drying process [5]. Orthodox seeds undergo mature dehydration on the maternal plants. Their water content is usually low and can be further dried to 1 to 5% moisture without damage [4,6].

LEA was first isolated from cotyledons of cotton at the late embryonic development stage [7]. In the following 30 years, LEA proteins have been detected in a variety of higher plant development seeds [8,9,10,11,12,13]. The LEA family is usually composed of multiple members in plants. Previous study identified at least 13 and 34 *LEA* homologous genes in maize [14] and rice, respectively [15]. With the development of genome-wide sequencing technology, the *LEA* gene family has been further elucidated in many other species, such as *Solanum lycopersicum* (27 *LEA* genes) [16], *Dendrobium officinale (Orchidaceae)* (17) [17], *Brassica mapus* (108) [18], Amaranth (105) [19], and *Solanum Tuberosum* (74) [13]. Furthermore, *LEA* genes are not unique to plants. They have been also identified in fungi, bacteria and animals [20].

LEA proteins have small molecular weights from 10 to 30 kDa. They are enriched in glycine and other hydrophilic amino acids, resulting in high hydrophilicity and thermal stability [21]. LEA proteins consist of a non-periodic linear and facultative α-helix which formed the main hydrophobic interaction between monomers without thermodynamically dominant state [8,22]. According to the similarity, LEA family is generally divided into 8 groups, namely LEA1, LEA2, LEA3, LEA4, LEA5, LEA6, dehydrin and SMP (seed maturation protein) [23]. Recently, ASR proteins (abscisic stress ripening) were classified as LEA7 group [24,25], extending LEA family to 9 groups. The division of family members is not consistent in different species [26]. While the sequence conservation implies their conserved role in desiccation tolerance [27].

Moreover, LEA proteins were reported to be widely involved in plant stress response in Arabidopsis, tobacco, grape, and other species [28]. In monocotyledons such as maize, wheat and rice, LEA expression can be induced by varieties of abiotic stresses to improve plant tolerance [29,30,31], which is beyond the scope of this article.

Wheat, as an important staple crop in the world, is becoming the focus of global food security issues [32,33]. In China, due to changes of climate and cultivation methods, new requirements for early maturing traits and dehydration characteristics of winter wheat have been put forward recently [34,35]. The moisture content of wheat seeds, like orthodox seeds, decline up to 40% quickly after physiological ripening. The speed of grain dehydration affects the desiccation tolerance and is highly related to the harvest time after maturity. Rapid dehydration after maturity is able to avoid the bad meteorological conditions in late stages of seed maturation, reduce the occurrence of pre-harvest sprouting, and shorten the cost of drying after harvest.

Wheat is an allohexaploid species with a large and complex genome consisting of three closely related sub genomes (A, B, and D). The continuous improvement and release of genomic data promoted the identification and analysis of wheat genes genome-widely [36,37,38,39,40,41,42]. The identification and use of the elite gene resource may help us improve the stress tolerance of wheat and solve the problems caused by changing global climate and food security status [42].

In this study, we systematically characterized the LEA genes in wheat based on the latest genome sequences. The genome composition, phylogeny, conserved motifs, chromosome locations, and the expression pattern of TaLEA proteins were systematically analyzed. These results will not only help us to reveal the molecular mechanism of mature dehydration in wheat and other cereal crops, but also provide a theoretical basis for their application to promote crop variety improvement.

## 2. Materials and Methods

### 2.1. Identification and Phylogenetic Analysis of Late Embryogenesis-Abundant (LEA) Genes in Wheat Genomes

The nucleotide and protein sequence in the whole-genome of *T. aestivum* was downloaded from the wheat genome URGI database (https://wheat-urgi.versailles.inra.fr/Seq-Repository). These sequences were first used to construct a local protein database with which to search against known LEA protein sequences collected from *A. thaliana* (51) [9] and *O. sativa* (34) [15], through a local protein basic local alignment search (BLASTP) program (https://blast.ncbi.nlm.nih.gov) with an E-value cut-off <10^−10^ and an identity of 50% as the threshold. The Hidden Markov Model (HMM) which was constructed using HMMER 3.0 program was used to search all the encoding protein sequences of wheat to find all predicted LEA family members. A self-blast of these sequences was performed by comparing of HMMER and BLAST hits to remove the redundancy, and no alternative splice variants were considered. The aligned sequences were then used as candidate LEA family sequences. The obtained candidate sequences were subjected to domain annotation of the target sequence using the software PfamScan and Pfam A databases, and the sequences containing the complete LEA-1 (Pfam ID PF03760.14), LEA-2 (PF03168.12), LEA-3 (PF03242.12), LEA-4 (PF02987.15), LEA-5 (PF00477.16), LEA-6 (PF10714.8), Dehydrin (PF00257.18), and SMP (PF04927.11) domains were identified as the final wheat LEA family sequences. The identified wheat LEA proteins were termed according to the subfamily it belongs and the order of gene ID. The biochemical parameters such as theoretical isoelectric point (PI), molecular weight (MW), amino acid number, instability, and hydrophilicity of the obtained proteins were calculated by the ProtParam (http://web.expasy.org/protparam/). To investigate the evolutionary relationships among these *TaLEA* genes, multiple sequence alignment was performed using MAFFT (Multiple Alignment using Fast Fourier Transform: http://mafft.cbrc.jp/alignment/software/) with the default parameters. A tree was constructed based on the full-length protein sequences using the Neighbor-Joining (NJ) method with Partial deletion and p-distance model, and a Bootstrap test of 1000 replicates for internal branch reliability. Another two phylogenetic trees for LEA protein family of *T. aestivum*, *O. sativa*, and *A. thaliana* were generated using Maximum-Likehood [ML, by FastTree (an open-source software developed by Morgan N. Price in Adam Arkin’s group at Lawrence Berkeley National Lab, Berkeley, USA) with default parameters] and Maximum-Parsimony (MP, by MEGA7.0 software [43] with the Max No. of Trees to Retain as 100) method to better understand the evolution of LEA genes.

### 2.2. Structural Characterisation of Wheat LEA

The exon-intron structures of the *TaLEA* family genes were determined based on alignments of their coding sequences with the corresponding genomic sequences, and a diagram was obtained using GSDS (Gene structure display server: http://gsds.cbi.pku.edu.cn/). To better understand the similarity and diversity of protein motifs, MEME (Multiple Expectation Maximization for Motif Elicitation: http://meme.nbcr.net/meme) was used to identify the conserved motif structures encoded by each group of *TaLEA* family genes. The parameters as follow: optimum motif widths of 6–50 residues and a maximum of 15 motifs. The schematic diagram of the amino acid motifs for each *TaLEA* gene was drawn accordingly.

### 2.3. Distribution of LEA Genes on Wheat Chromosomes

*TaLEA* were mapped on wheat chromosomes according to the positional information of the *TaLEA* genes from the wheat genome annotation datebase, and the chromosome physical location map was displayed using MG2C (Map Gene 2 Chromosome V2: http://mg2c.iask.in/mg2c_v2.0/).

### 2.4. Expression Profile Analysis of Wheat LEA Genes

The publicly available wheat RNA-Seq datasets used for generating gene expression levels were downloaded from the Wheat Expression Browser (http://www.wheat-expression.com), then used to analyze the expression profiles of the identified *TaLEA* genes. These data are from the studies of developmental time-course of common hexaploid wheat variety Azhurnaya and Chinese Spring. The results were summarized according to high expression level tissues as grains, roots, leaves/shoots, and spikes. The original TPM (transcripts per kilobase million) of each gene plus 0.0001 to prevent an expression value of ‘0’. The heat map was drawn in Log_10_ (tpm + 0.0001)-transformed expression values according to the standardization. Genes highly expressed in the grain were selected for qRT-PCR detection.

### 2.5. Plant Materials, Growth Conditions, and Sampling

Based on many years production experience and research, Yangmai16 had a faster grain filling speed, earlier maturity compared with Yangmai15 [35]. Therefore, these two spring wheat varieties Yangmai16 and Yangmai15, which were bred by the author’s unit, were planted and cultivated under the same condition. At the flowering stage, two individual florets at the base of the center ears were marked with a marker pen and the plants were tagged with anthesis dates. Wheat grains were sampled at 10, 15, 20, 25, 30, and 35 DAA (days after anthesis) for extraction of total RNA and further determination.

### 2.6. Quantitative Real-Time Polymerase Chain Reaction Analysis of TaLEA Proteins

In order to identify the expression profile of *TaLEA* gene in different wheat cultivars, and to explain the relationship between the LEA protein and maturity, as well as the grain production, high expression level LEA genes were collected for quantitative real-time polymerase chain reaction (qRT-PCR) analysis. Total RNA was extracted from the seeds by Trizol reagent (Biotech, Shanghai), and the first strand of cDNA was synthesized using Transcriptor First Strand cDNA Synthesis Kit (Roche, Penzberg, Germany). The wheat gene *Actin* was used as an internal reference, and the selected 12 *TaLEA* genes, including *TaDehydrin-1*, *TaDehydrin-3*, *TaDehydrin-5*, *TaDehydrin-7*, *TaLEA1-1*, *TaLEA1-3*, *TaLEA4-13*, *TaLEA5-1*, *TaLEA5-3*, *TaLEA5-4*, *TaSMP-1* and *TaSMP-2*, were subjected to qRT-PCR using a fluorescence quantitative reagent Ultra SYBR Mixture (Kang Wei Century, Beijing) on a Roch Light-Cycler 480 fluorescence quantitative PCR machine (Roche, Penzberg, Germany); three independent biological replicates. The primer sequences used are listed in Table S1.

### 2.7. Determination of the Dry Weight and Moisture Content of Grains

Sampled grains were used for measurements of grain dry weight and moisture content. The fresh grains then dried at 70 °C to constant weight were weighed for the fresh weight (*W_F_*) and dry weight (*W_D_*). Moisture content of grains was calculated by (*W_F_* − *W_D_)*/*W_F_* × 100%.

The processes of grain filling were fitted by Logistic’ equation as described by [44]:

f(x) = a/(1 + b × exp(−c × x))(1)

The dehydration progress was fitted by Gompertz equation as described by [45]:

f(x) = a × exp(−exp(−b × (x − c)))(2)

## 3. Results

### 3.1. Genome-Wide Identification and Phylogenetic Analysis of Wheat LEA Genes

Based on genome-wide blast, a total of 121 non-redundant *LEA* homologous containing the complete LEA, Dehydrin, and SMP domains were identified from the hexaploid wheat genome, which were considered as the putative wheat *LEA* genes. By phylogenetic analysis, these 121 genes were divided into 8 groups (groups *LEA1*–*LEA6*, *Dehydrin*, and *SMP*) (Table 1). The largest group *Dehydrin* contains 47 members, while the smallest groups *LEA3* and *LEA6* have only two members. Groups *LEA1*, *LEA2*, *LEA4*, *LEA5,* and *SMP* contain 19, 15, 15, 4, and 17 genes, respectively.

The physical and chemical parameters of 121 TaLEA proteins were shown according to the analysis using the ProtParam online tool. The wheat LEA protein has a maximum length of 457 amino acid residues (TaDehydrin-8) and the shortest of 99 amino acid residues (TaLEA3-2), with isoelectric points (pI values) ranged from 4.18 (TaSMP-15) to 10.74 (TaDehydrin-28). The molecular weights range from 10.64 kDa (TaLEA3-2) to 43.89 kDa (TaDehydrin-8) with an average of 20.98 kDa. Only 16 proteins have a molecular weight >30 kDa. Analysis of the grand average of hydropathicity (GRAVY) index indicated that all TaLEA proteins were hydrophilic, with all of the index <0. The lipid index reflects the thermal stability of the protein in Table 1.

**Figure 1:**
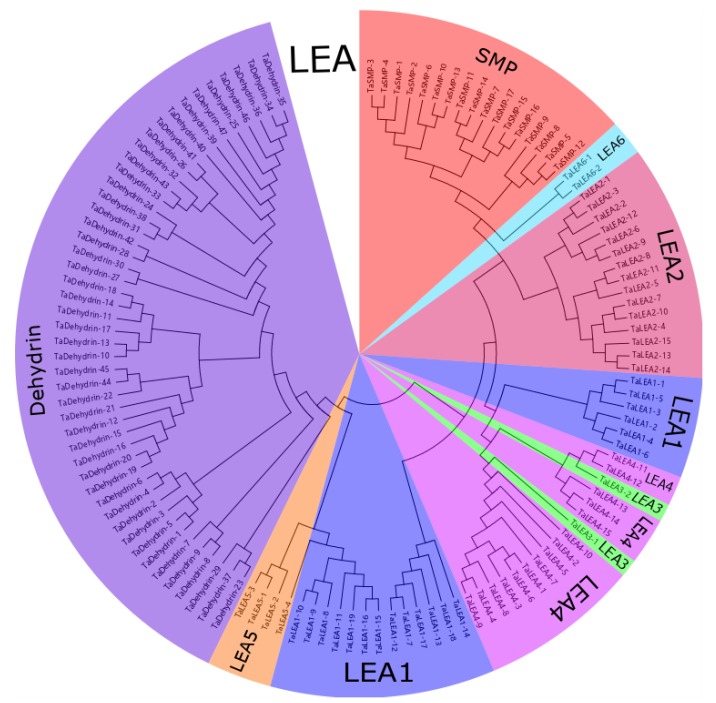
Phylogenetic analysis of wheat Late Embryogenesis-abundant (LEA) proteins. The LEA1, LEA2, LEA3, LEA4, LEA5, LEA6, Dehydrin, and seed maturation protein (SMP) groups are presented in blue, pink, light green, bright purple, orange, light blue, purple, and red, respectively.

The phylogenetic analysis (Figure 1) revealed two major clades of the *TaLEA* family. The 47 genes of the *TaDehydrin* group, the LEA5 group, and part of the LEA1 group were clustered in one clade, while the other six groups were clustered in another one. According to the evolutionary relationship, *TaSMP* is more closely related to LEA6 and LEA2; LEA1 and LEA4 are more closely related. To classify the *TaLEA* genes into different subfamilies with higher confidence and to better understand their evolutionary relationship, another two phylogenetic trees were constructed with *Arabidopsis* and *Oryza* (Figures S1 and S2). It was indicated that the subfamilies classification of *LEA* genes in wheat is credible.

### 3.2. Structural Characterization of Wheat LEA

**Figure 2:**
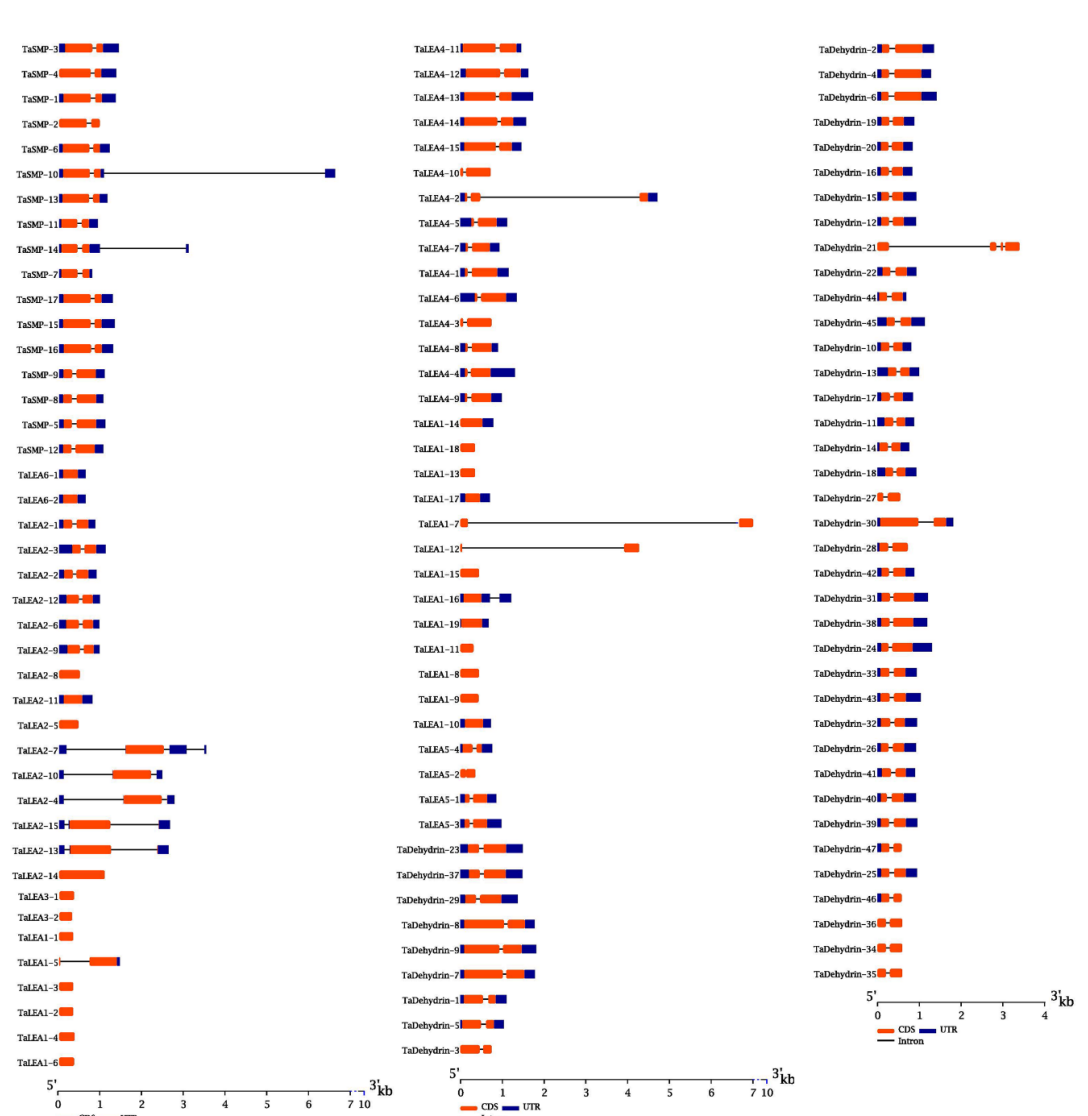
Exon-intron structures of *TaLEA* genes. Exon-intron are indicated by wide color bar and black line, respectively.

All Gene structure analysis revealed that (Figure 2) wheat LEA genes generally contained few introns, and 24 had no introns. Only two of the 97 intron-containing LEA proteins contained more than one intron. Among the 47 genes of the *TaDehydrin* group, 46 genes contained only one intron, while the other one (*TaDehydrin-21*) contained three introns. In the *TaLEA1* group, 16 genes had no introns, and the other 3 genes contained only one intron. Among the 15 genes in the *TaLEA2* group, 11 were single-intron genes, and the other 4 were intron-free genes. Neither *LEA3* nor *LEA6* contained introns. There were 14 single-intron genes and 1 double intron gene among the *TaLEA4* group. Both of the *TaLEA5* and *TaSMP* groups contained only one intron.

**Figure 3:**
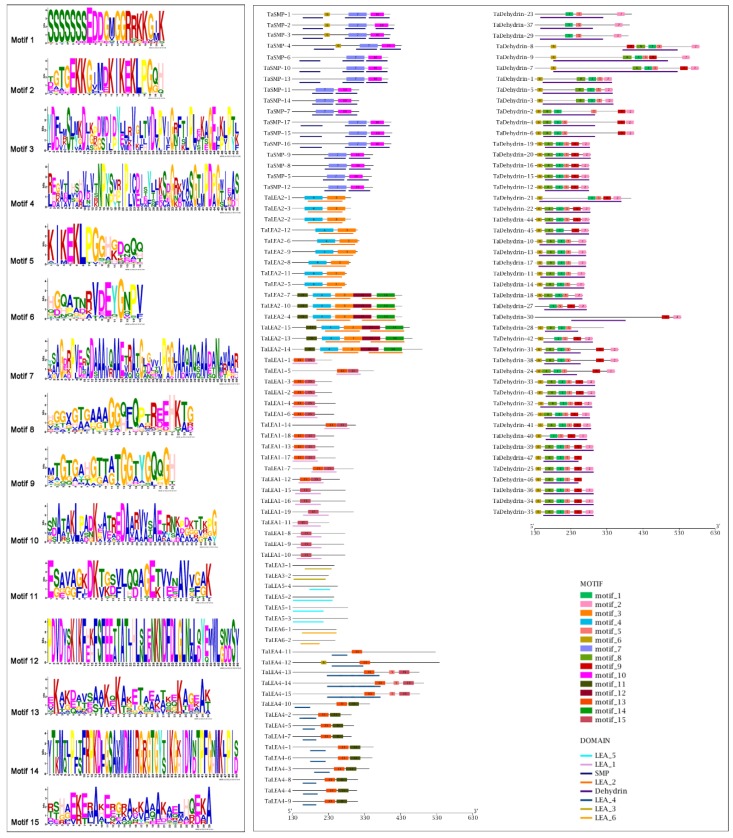
Conserved motifs (**left**) and their distributions (**right**) of *TaLEA* genes predicted by the MEME (Multiple Expectation Maximization for Motif Elicitation) online tool. Each motif is represented by a different colored box with corresponding number. The domain of each subfamily is represented by a different colored thick line below.

To elucidate the similarity and diversity of protein motifs, the conserved motifs of wheat LEA proteins in each group were analyzed by MEME software. 15 conserved motifs were identified, namely motif1~motif15 (Figure 3). The results indicated that with the exception of the TaLEA3, TaLEA5, and TaLEA6, there were conservative motifs specific to each group (Figure 3). The composition of structural motifs was diverse among different LEA groups, but similar within the same group. Moreover, the motifs encoding LEA domains were relatively conserved, indicating that the functions of TaLEA proteins are intergroup specific. All of the 19 genes in the LEA1 group have motif15, and 12 of them also contain motif13. Among the LEA2 group, 9 members contained 2 motifs and 6 members contained 5 motifs, while each member possessed motif3 and motif4. The TaSMP group contained motif7 and motif10. All LEA4 members had motif13, with 10 of them also having motif11, and 3 of them having motif5 and motif15. There were 27 genes in the Dehydrin group possessing motif1, motif2, motif5, motif6, motif8, and motif9, and all 47 members contained motif1, motif2, and motif5.

### 3.3. Chromosomal Locations of Wheat LEA Genes

**Figure 4:**
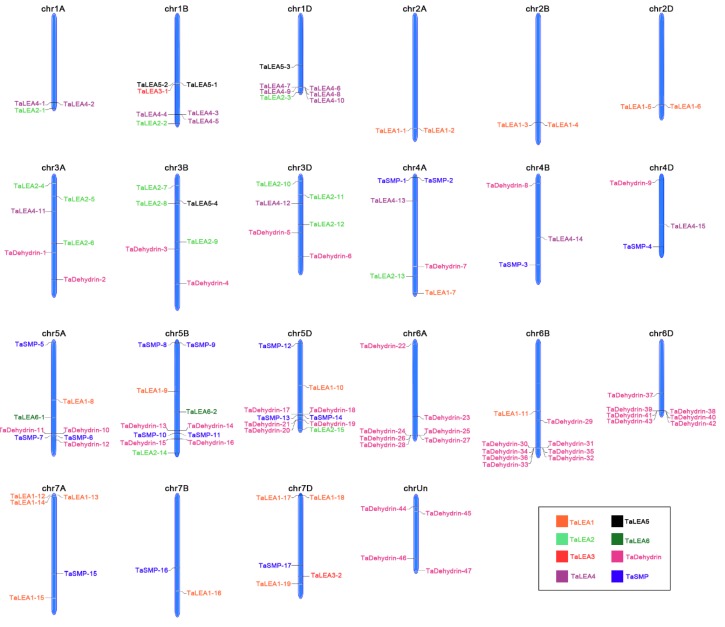
Distribution of *TaLEA* gene family members on wheat chromosomes. *TaLEA1*, *TaLEA2*, *TaLEA3*, *TaLEA4*, *TaLEA5*, *TaLEA6*, *TaDehydrin* and *TaSMP* subfamily are presented in orange, light green, red, purple, black, dark green, pink, and blue.

Using MapGene2Chrom software to analyze genomic position data, 117 genes were distributed on 21 wheat chromosomes, while the location of the other 4 genes remained unclear (Figure 4). The analysis from the 7 homologous chromosome groups showed that there were 29 *LEA* genes on the chromosome groups five, representing the most abundant regions, followed by group six with 23, while the minimum was on chromosome groups two, with only 6 genes. Chromosome groups one and three had 18 and 17 genes, respectively. Chromosome groups four and seven had 12 genes. In total, 37, 40, and 40 *TaLEA* genes were located on the A, B, and D sub-genome, respectively, which were almost evenly distributed.

According to the chromosomal distribution of the *TaLEA* gene, there were 43 of the *Dehydrin* group distributed on homologous chromosome groups three, four, five, and six, except for the four genes were still unclear. Among them, there were 7, 8, 7 on chromosome 6A, 6B, and 6D, and 3, 4, 5 on 5A, 5B, and 5D, respectively, accounting for 72.34% of all *Dehydrin* members. Such a concentrated distribution deserves further study in the future.

### 3.4. Expression Profile Analysis of Wheat LEA Genes in Different Tissues

**Figure 5:**
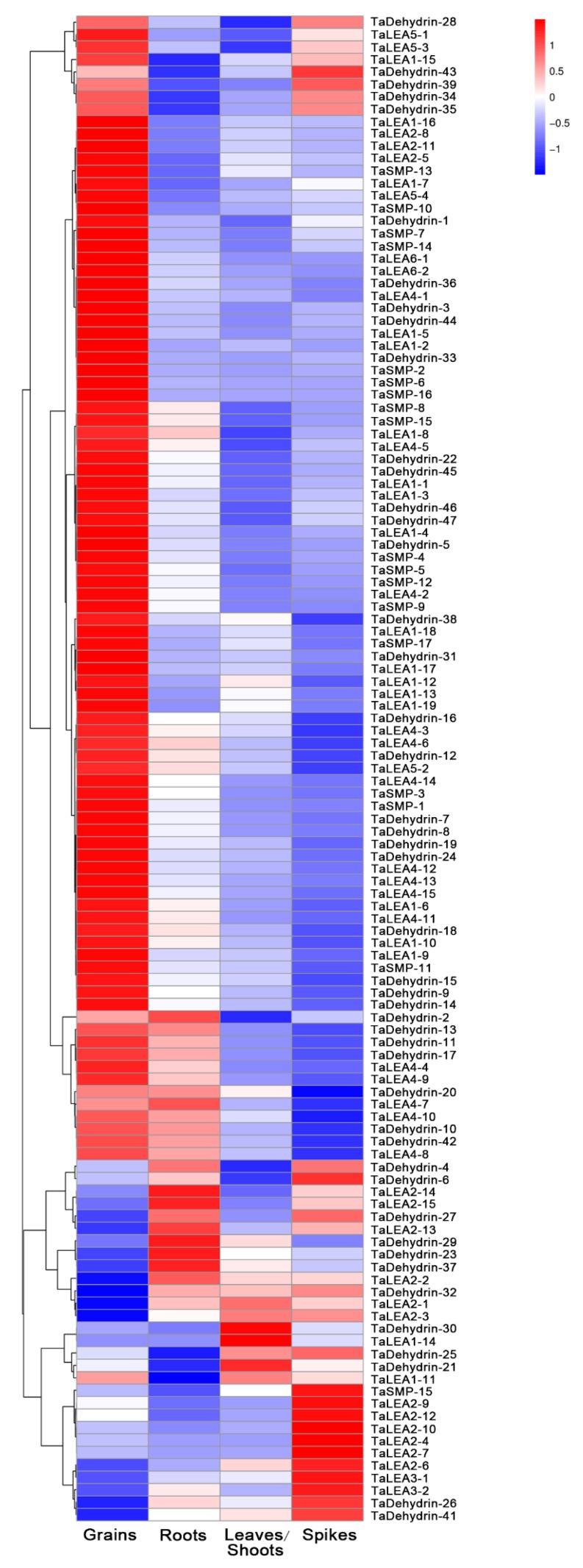
Expression profiling of *TaLEA* genes in different tissues. RNA-seq date downloaded from Wheat Expression Browser was used to analysis expression pattern. The heat map was drawn in Log_10_ (tpm + 0.0001)-transformed expression values. Color scale on the right represent relative expression levels: red represents high level and blue represent low level.

To gain insight about the expression profile of these identified *TaLEA* genes, the publicly available RNA-Seq data were collected from the Wheat Expression Browser database. The expression analysis was performed on grains, roots, leaves/shoots, and spikes. A heat map was created according to the Log_10_ (tpm + 0.0001) of 121 *TaLEA* genes (Figure 5). It was indicated that expression patterns of *TaLEA* genes in different tissues were quite different. 93 genes highly expressed in grains, and among them there were 35 out of 47 members in the Dehydrin subfamily; 17 out of 19 in LEA1; all members of LEA4, LEA5 and LEA6. Whereas, very low expression levels of these 93 genes were observed in the other three tissues, except for 12 in roots and 8 in spikes were also high. 11 highly expressed genes were only observed in spikes, and among them there were 6 members of LEA2 and two members of the LEA3 subfamily. Almost no gene highly expressed in all four tissues and only several genes had higher expression levels in multiple tissues.

### 3.5. qRT-PCR Analysis of Wheat LEA Genes in the Grain Maturation of Two Different Cultivers

According to the analysis of gene expression in different tissues, 12 *TaLEA* genes highly expressed in grains were selected to verify the potential role of *TaLEA* genes in the maturation of seed. Expression levels at different stages post anthesis of Yangmai16 and Yangmai15 were analyzed by qRT-PCR.

**Figure 6:**
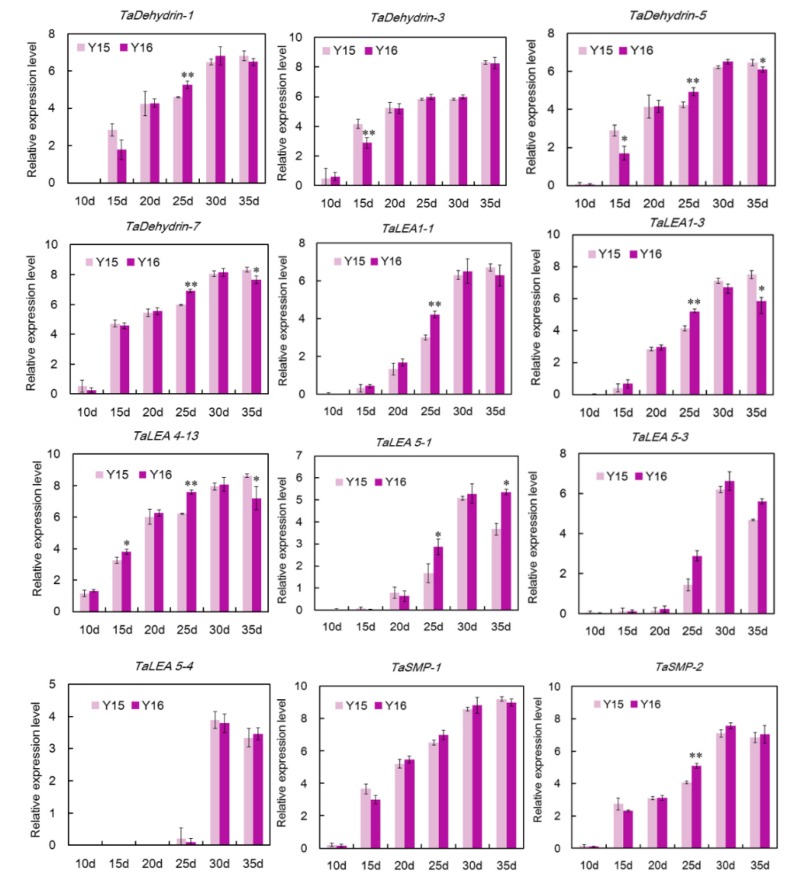
Expression profiles of 12 wheat *LEA* genes during grain filling and maturation. Purple and light purple columns indicate Yangmai16 and Yangmai 15, respectively. Values represent means ± standard deviation of three replicates. Asterisks reveal the gene significantly higher or lower in Yangmai 16 than in Yangmai 15 by *t*-test (* *p* < 0.05, ** *p* < 0.01).

Results showed that the expression of these *TaLEA* genes have temporal specificity. On the whole, the expression level of *TaLEA* genes increased from 15 DAA to 35 DAA (Figure 6) in two genotypes. Interestingly, the expression patterns of *TaLEA* genes in two genotypes presented different characteristics. In Yangmai16, the maximum expression level of 9 genes except *TaDehydrin-3*, *TaLEA5-1* and *TaSMP-1* were observed at 30 DAA, whereas there were 8 genes in Yangmai 15 with maximum expression level at 35 DAA. For most genes, the peak of expression appeared earlier in Yangmai16. Statistical analysis indicated that, at 25 DAA, the expression level of 8 genes in Yangmai 16 was significantly higher than Yangmai 15.

**Figure 7:**
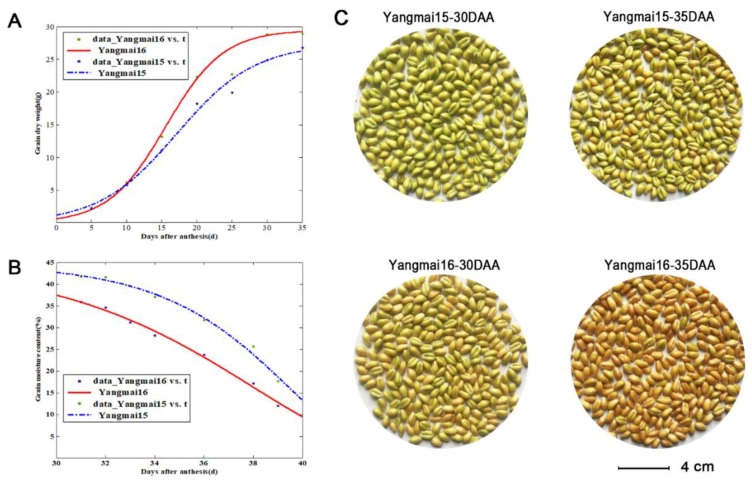
Phenotypic character of grain maturation in Yangmai 15 and Yangmai 16. (**A**), comparison of grain dry weight. (**B**), comparison of grain moisture content. (**C**), appearance of grains during maturation progress; Bar = 4 cm.

According to the determination of phenotypic character of maturation, the increase of grain dry weight in Yangmai16 was faster than that in Yangmai15 (Figure 7A). At 30 DAA, the grain filling of yangmai16 grain was almost completed. Statistical analysis indicated that the average grain filling rate was significantly higher in Yangmai 16 (*P* = 0.0185). The moisture content curve and the average seed dehydration rate indicated that the dehydration of Yangmai 16 was faster (*P* = 1.69 × 10^−4^) and started earlier than Yangmai 15, which could be judged by the appearance and color of the seeds (Figure 7B,C).

## 4. Discussion

### 4.1. Identification and Analysis of Wheat LEA Gene Family

In this study, 121 wheat *LEA* homologous were identified by genomic analysis. Wheat LEA family is bigger than rice (34), corn (32), soybean (36), potato (74), Arabidopsis (51), and canola (108), but less than upland cotton (242). We speculated that in the process of hybridization and chromosome doubling by three species, the number of genes had also doubled and finally formed an allohexaploid species with a huge genome [46,47,48]. It is also possible that most of the wheat LEA genes were retained during subsequent evolution due to genome replication [49]. Functional differentiation of *LEA* genes during the evolution process could facilitate the regulation of development and adaptation to the environment.

The largest group of the wheat LEA family Dehydrin accounts for about 40% of the total number of family members. However, the largest group was Dehydrin in rice [15] and LEA3 in soybeans [50]. In plum blossom, the largest groups were Dehydrin and LEA2 [10]. LEA2 group in potato had the most members, accounting for 45 of all 74 LEA genes, while the Dehydrin group had only 5 [13]. The difference above indicated that *LEA* family genes may have evolved independently after the differentiation of these species.

Gene structure analysis revealed that wheat LEA family genes generally lack introns. For instance, there were 24 genes that don’t have introns and 95 genes that have only one intron, while only 2 genes contained more than one intron. The phenomenon was also found in other species [13,50,51]. Perhaps it could be explained by the early intron model of protein-coding gene origin [52]. In general, genes that respond to stress contain fewer introns. Introns can have a deleterious effect on gene expression by delaying transcript production [18]. Moreover, introns can extend the length of the nascent transcript, resulting in an additional expense for transcription [53]. The small number of introns may be a result of adaptation to the environment, which takes less time from transcription to translation, allowing rapid gene expression and functional protein production in response to stresses [53]. DREB [54], AP2/ERF [55], and HD-Zip [42], which play important roles in response to diverse stresses, showing similar phenomenon. 

The expression profiles of *TaLEA* family members in different tissues suggested that many *TaLEA genes* have obvious spatial specificity. However, they were still mainly enriched in the spikes and grains. Our study could be helpful for further investigation of *LEA* genes with spatiotemporal expression specificity and evaluation of their roles in desiccation tolerance.

### 4.2. Wheat Seed Desiccation and the Roles of TaLEA Genes in Acquirement of Desiccation Tolerance

With the prospect of climate changes that are shaping future agricultural practices, the production of highly vigorous seeds have become a key lever to improve yields and a focus of food security and genetic resources [56]. Because of the long growth duration of common wheat varieties, plants must face various biotic and abiotic stresses in fields, especially during the stage of seed maturity [57]. The stage of seed maturity at harvest, which was also associated with the desiccation tolerance, is a major factor influencing seed vigor and grain quality [58]. Nowadays, faster filling and dehydration of wheat seeds achieved more popularity in China because of the important role for optimizing the new cropping systems.

The wheat seeds undergo three development phases: embryo morphogenesis, material accumulation, and mature dehydration. During the last stage, the metabolism is correspondingly weakened, along with the rapid decrease of water content, which then turn on the transition from morphogenesis to germination [59,60,61]. Before and during dehydration, physiological and biochemical changes contribute to the acquisition of desiccation tolerance.

The correlation between LEA protein and desiccation tolerance had been confirmed in many orthodox seeds [62,63]. In this study, the spatial expression pattern of *TaLEA* indicated that some genes display high expression levels mainly in spikes and grains. Two spring wheat variety Yangmai16 and Yangmai15 were then used for quantitative verification. The results indicated that Yangmai16 had certain advantages in time and quantity of expression at 25 DAA, in which the maximum expression of 8 *TaLEA* genes appeared earlier than that of Yangmai15. It could be a hint of the inherent genetic differences between two varieties. We speculated that the phenotype of earlier and faster dehydration, which may also possess higher desiccation tolerance theoretically, were correlated with the different expression levels of *LEA* genes in grains.

The contribution of LEA to maintain seed desiccation tolerance is possibility due to their thermal stability and high hydrophilicity, acting as a dehydration protectant and substitute of water [64]. During the mature dehydration of seeds, the protein of Dehydrins will be present in the cell like a “space filler”, maintaining the dissolved character of cell fluid to avoid damage to cell architecture [21]. Magnetic Resonance Imaging (MRI) has been used for tracking and locating the distribution regular of water during seed maturation. It was shown that under the action of Dehydrins, water moves against potential, and the sufficient water in the surroundings of the seed could maintain the available flowing water content in the cells [65,66]. LEA were also supposed to regulate the expression of other genes by direct binding, which still lacks evidence.

Four questions that need to be considered were raised by Song and Fu in the last century [67]: What are the signals that induce the mature dehydration during seed development? Are these signals from the seed itself (embryo, storage tissue, and seed coat) or the maternal tissues? What are the acceptors in seeds that response to the dehydration signal? And last but not least, what are the subtle effects of LEA proteins in cells and sub-cells leading to desiccation tolerance? Answers to these questions would be valuable for a better understanding of mature dehydration mechanisms and germplasm conservation, as well as the genetic improvement of crops.

## Figures and Tables

**Table 1:** Description of Late Embryogenesis Abundant (*LEA*) genes identified from the wheat genome.

Gene_Name	Gene_ID	Amino Acid Number	MW	pI	Instability Index	Aliphatic Index	GRAVY
TaDehydrin-1	TraesCS3A02G254600	213	21,831.76	6.25	35.63	46.76	−0.83
TaDehydrin-10	TraesCS5A02G369800	143	14,570.81	8	34.57	36.36	−1.151
TaDehydrin-11	TraesCS5A02G369900	140	14,240.47	8.01	38	39.86	−1.083
TaDehydrin-12	TraesCS5A02G424800	149	15,217.65	9.33	25.94	32.21	−1.087
TaDehydrin-13	TraesCS5B02G372100	143	14,429.64	8	34.24	35.66	−1.118
TaDehydrin-14	TraesCS5B02G372200	138	14,218.46	8.01	38.03	38.99	−1.104
TaDehydrin-15	TraesCS5B02G426700	150	15,176.65	9.52	31.65	34.6	−1.062
TaDehydrin-16	TraesCS5B02G426800	150	15,221.65	9.36	24.14	32	−1.063
TaDehydrin-17	TraesCS5D02G379200	143	14,515.73	7.11	33.97	36.99	−1.123
TaDehydrin-18	TraesCS5D02G379300	133	13,930.2	8.81	40.1	38.95	−1.189
TaDehydrin-19	TraesCS5D02G433200	152	15,345.79	9.52	27.66	32.24	−1.05
TaDehydrin-2	TraesCS3A02G396200	275	27,016.77	9.6	26.32	37.13	−0.775
TaDehydrin-20	TraesCS5D02G433300	154	15,587.09	9.52	25.9	31.17	−1.024
TaDehydrin-21	TraesCS5D02G519300LC	266	28,166.53	9.76	46.85	52.93	−0.803
TaDehydrin-22	TraesCS6A02G059800	153	15,511.87	8.84	37	39.08	−1.041
TaDehydrin-23	TraesCS6A02G253300	268	28,823.05	5.25	56.43	63.73	−0.997
TaDehydrin-24	TraesCS6A02G350500	221	22,048.66	9.05	7.59	23.48	−1.071
TaDehydrin-25	TraesCS6A02G350600	162	16,284.68	9.22	17.14	38.02	−0.994
TaDehydrin-26	TraesCS6A02G350700	152	15,522.71	7.17	27.53	31.51	−1.176
TaDehydrin-27	TraesCS6A02G350800	143	14,815.21	9.22	37.6	36.36	−1.012
TaDehydrin-28	TraesCS6A02G350900	190	20,142.87	10.74	50.46	35.63	−1.274
TaDehydrin-29	TraesCS6B02G273400	259	27,973.07	5.2	57.39	62.93	−1.054
TaDehydrin-3	TraesCS3B02G286600	217	22,297.38	6.87	35.47	47.7	−0.856
TaDehydrin-30	TraesCS6B02G383200	405	40,293.09	6.83	-0.26	29.98	−1.064
TaDehydrin-31	TraesCS6B02G383500	231	23,229.05	9.22	2.83	23.25	−1.074
TaDehydrin-32	TraesCS6B02G383600	158	15,838.17	9.13	23.2	34.11	−1.041
TaDehydrin-33	TraesCS6B02G383800	166	16,704.04	8.05	17.71	33.01	−1.054
TaDehydrin-34	TraesCS6B02G695700LC	162	16,127.46	9.19	13.99	35	−0.983
TaDehydrin-35	TraesCS6B02G695800LC	162	16,127.46	9.19	13.99	35	−0.983
TaDehydrin-36	TraesCS6B02G695900LC	162	16,097.44	9.19	14.98	35.62	−0.968
TaDehydrin-37	TraesCS6D02G234700	262	28,155.2	5.19	57.34	61.83	−1.051
TaDehydrin-38	TraesCS6D02G332900	231	23,020.75	9.05	6.9	24.16	−1.028
TaDehydrin-39	TraesCS6D02G333000	162	16,195.51	8.05	14.92	33.21	−1.019
TaDehydrin-4	TraesCS3B02G428200	274	27,193.96	9.74	24.64	34.34	−0.859
TaDehydrin-40	TraesCS6D02G333100	144	14,512.72	9.19	15.79	33.96	−1.076
TaDehydrin-41	TraesCS6D02G333200	155	15,729	8.83	26.81	34.06	−1.125
TaDehydrin-42	TraesCS6D02G333300	160	16,255.59	8.07	21.61	39.81	−1.05
TaDehydrin-43	TraesCS6D02G333600	167	16,712.02	7.17	14.3	31.08	−1.062
TaDehydrin-44	TraesCSU02G086200	151	15,288.66	9.1	38.15	42.19	−1.011
TaDehydrin-45	TraesCSU02G122200	149	14,855.02	6.86	33.61	40.13	−0.947
TaDehydrin-46	TraesCSU02G564500LC	130	12,865.8	9.05	25.25	32.38	−0.968
TaDehydrin-47	TraesCSU02G656800LC	130	12,865.8	9.05	25.25	32.38	−0.968
TaDehydrin-5	TraesCS3D02G255500	215	22,243.3	6.63	37.53	44.51	−0.912
TaDehydrin-6	TraesCS3D02G390200	275	27,156.85	9.7	23.91	34.58	−0.855
TaDehydrin-7	TraesCS4A02G250900	455	43,740.8	8.84	-6.02	31.38	−0.779
TaDehydrin-8	TraesCS4B02G064200	457	43,891.98	8.84	-3.84	32.74	−0.741
TaDehydrin-9	TraesCS4D02G063100	430	41,223.14	9.04	0.85	31.65	−0.773
TaLEA1-1	TraesCS2A02G449700	110	11,589.91	9.4	32.94	39.45	−1.056
TaLEA1-10	TraesCS5D02G177300	146	14,589.98	7.11	25.53	57.81	−0.588
TaLEA1-11	TraesCS6B02G244900	102	10,650.63	8.06	34.58	42.55	−1.089
TaLEA1-12	TraesCS7A02G030100	131	13,910.81	9.87	39.53	54.66	−0.852
TaLEA1-13	TraesCS7A02G030300	115	12,294.95	9.84	38.36	58	−0.886
TaLEA1-14	TraesCS7A02G042500	175	19,508.1	10.39	47.89	52.23	−1.061
TaLEA1-15	TraesCS7A02G439200	147	14,483	9.13	30.34	57.48	−0.453
TaLEA1-16	TraesCS7B02G337800	147	14,522.94	8.93	30.4	51.5	−0.555
TaLEA1-17	TraesCS7D02G026300	119	12,561.2	9.84	33.94	56.13	−0.845
TaLEA1-18	TraesCS7D02G026400	115	12,306.88	9.94	29.52	52.96	−0.95
TaLEA1-19	TraesCS7D02G428800	169	16,768.61	9.56	24.28	55.86	−0.442
TaLEA1-2	TraesCS2A02G449800	110	11,406.59	9.19	15.59	36.82	−1.047
TaLEA1-3	TraesCS2B02G471500	110	11,481.72	8.69	28.51	39.45	−0.993
TaLEA1-4	TraesCS2B02G471600	120	12,363.79	9.58	25.19	37.83	−0.96
TaLEA1-5	TraesCS2D02G449200	226	24,041.95	10.1	51.53	45.66	−0.828
TaLEA1-6	TraesCS2D02G449300	116	12,059.47	9.58	21.73	40.78	−0.975
TaLEA1-7	TraesCS4A02G459700	169	18,344.61	10.03	45.86	55.15	−0.931
TaLEA1-8	TraesCS5A02G172800	146	14,712.07	6.71	25.02	53.15	−0.634
TaLEA1-9	TraesCS5B02G170200	143	14,466.79	6.65	24.27	54.34	−0.645
TaLEA2-1	TraesCS1A02G423800	163	17,826.52	5.03	14.94	89.75	−0.069
TaLEA2-10	TraesCS3D02G091400	305	33,630.14	4.65	28.86	92.98	−0.329
TaLEA2-11	TraesCS3D02G158600	151	16,272.66	4.78	14.24	93.58	−0.068
TaLEA2-12	TraesCS3D02G227800	181	19,544.98	4.29	26.07	80.17	−0.173
TaLEA2-13	TraesCS4A02G343300	333	36,961.06	5.09	23.57	90.66	−0.393
TaLEA2-14	TraesCS5B02G531400	361	40,184.79	4.96	28.8	95.76	−0.288
TaLEA2-15	TraesCS5D02G529700	326	36,210.08	4.9	28.44	91.13	−0.406
TaLEA2-2	TraesCS1B02G455900	163	17,748.48	5.17	17.63	92.76	−0.022
TaLEA2-3	TraesCS1D02G432400	163	17,808.55	5.05	15.34	90.98	−0.037
TaLEA2-4	TraesCS3A02G091500	305	33,629.19	4.65	24.8	94.89	−0.297
TaLEA2-5	TraesCS3A02G150800	151	16,254.62	4.78	11.33	96.16	−0.055
TaLEA2-6	TraesCS3A02G225600	186	19,778.21	4.37	28.98	76.45	−0.165
TaLEA2-7	TraesCS3B02G106700	305	33,625.18	4.68	26.92	92.66	−0.343
TaLEA2-8	TraesCS3B02G240900LC	162	17,614.16	5.68	23.58	90.25	−0.199
TaLEA2-9	TraesCS3B02G255100	181	19,458.89	4.33	26.15	79.12	−0.169
TaLEA3-1	TraesCS1B02G243600	115	12,671.65	9.18	54.99	73.91	−0.42
TaLEA3-2	TraesCS7D02G389300	99	10,643.23	10.11	52.88	76.26	−0.167
TaLEA4-1	TraesCS1A02G364000	224	23,196.17	8.81	22.88	37.46	−1.118
TaLEA4-10	TraesCS1D02G369800	214	21,965.87	8.89	8.45	43.74	−0.95
TaLEA4-11	TraesCS3A02G188700	396	42,151.23	5.06	40.57	45.88	−0.962
TaLEA4-12	TraesCS3D02G192100	407	43,198.53	5.42	36.92	43	−0.963
TaLEA4-13	TraesCS4A02G129100	351	37,340.93	6.5	20.4	47.04	−1.007
TaLEA4-14	TraesCS4B02G175600	363	38,561.24	6.7	18.15	46.86	−1.031
TaLEA4-15	TraesCS4D02G177500	352	37,457.1	6.44	20.76	49.12	−0.97
TaLEA4-2	TraesCS1A02G364100	163	17,097.7	9.16	48.51	41.66	−1.045
TaLEA4-3	TraesCS1B02G381200	212	21,896.87	9.02	21.71	36.75	−1.096
TaLEA4-4	TraesCS1B02G381400	178	18,146.72	8.63	23.16	42.64	−0.954
TaLEA4-5	TraesCS1B02G381500	169	17,528	5.95	25.29	41.95	−1.009
TaLEA4-6	TraesCS1D02G369200	220	22,708.72	9	21.62	36.82	−1.108
TaLEA4-7	TraesCS1D02G369300	163	16,930.39	6.62	26.37	39.26	−1.026
TaLEA4-8	TraesCS1D02G369400	180	18,788.43	5.99	28.19	40.5	−1.046
TaLEA4-9	TraesCS1D02G369500	180	18,740.37	5.99	23.35	42.67	−1.029
TaLEA5-1	TraesCS1B02G237400	153	16,878.38	5.57	37.06	33.2	−1.539
TaLEA5-2	TraesCS1B02G436600LC	113	12,029.24	5.53	41.95	51.06	−0.962
TaLEA5-3	TraesCS1D02G225800	153	16,771.27	5.47	39.18	33.2	−1.503
TaLEA5-4	TraesCS3B02G166400	124	13,694.2	5.17	54.16	61.45	−1.308
TaLEA6-1	TraesCS5A02G258900	121	12,844.87	5.44	44.66	51.74	−1.065
TaLEA6-2	TraesCS5B02G257700	118	12,534.6	5.67	42.9	48.9	−1.044
TaSMP-1	TraesCS4A02G030600	272	27,106.72	4.72	35.79	70.59	−0.336
TaSMP-10	TraesCS5B02G390600	266	27,101.21	5.15	38.24	77.56	−0.316
TaSMP-11	TraesCS5B02G390800	185	18,756.74	4.69	20.34	75.24	−0.248
TaSMP-12	TraesCS5D02G026500	224	22,869.26	5.2	52.2	70.36	−0.442
TaSMP-13	TraesCS5D02G395600	266	27,028.16	5.04	38.1	79.4	−0.273
TaSMP-14	TraesCS5D02G395700	185	18,795.83	5.12	25.34	74.76	−0.258
TaSMP-15	TraesCS7A02G333100	277	28,312.94	4.18	23.46	70.04	−0.32
TaSMP-16	TraesCS7B02G244300	275	28,060.67	4.19	22.74	69.49	−0.308
TaSMP-17	TraesCS7D02G340400	275	28,179.74	4.19	20.55	68.76	−0.349
TaSMP-2	TraesCS4A02G030700	284	28,955.83	4.78	36.11	70	−0.412
TaSMP-3	TraesCS4B02G275300	272	27,037.57	4.65	34.14	69.89	−0.355
TaSMP-4	TraesCS4D02G273900	303	30,498.73	5.23	40.31	73.07	−0.305
TaSMP-5	TraesCS5A02G021400	220	22,346.74	5.31	50.58	71.64	−0.376
TaSMP-6	TraesCS5A02G385600	266	27,030.13	5.06	36.78	77.56	−0.306
TaSMP-7	TraesCS5A02G385700	185	18,762.78	5.21	21.47	74.16	−0.255
TaSMP-8	TraesCS5B02G018300	225	22,887.23	5.31	52.92	70.93	−0.424
TaSMP-9	TraesCS5B02G018900	224	23,131.59	5.17	43.42	72.1	−0.45

Note: MW, Molecular weight (Da); pI, Isoelectric point; GRAVY, Grand average of hydropathicity.

## Supplementary Materials

The following are available online at https://www.mdpi.com/2073-4425/10/9/696/s1, Table S1: Primers for qRT-PCR analysis; Figure S1: Phylogenetic analysis of LEA proteins among wheat (121), Arabidopsis (51) and rice (34) (by ML method); Figure S2: Phylogenetic analysis of LEA proteins among wheat (121), Arabidopsis (51) and rice (34) (by MP method).
