# Supplementary material for: Genome-Wide Identification and Expression Profiles of Late Embryogenesis-Abundant (LEA) Genes during Grain Maturation in Wheat (Triticum aestivum L.)
Source: Genes (Basel). 2019 Sep 10;10(9):696. doi: 10.3390/genes10090696 (PMC6770980; doi:10.3390/genes10090696)
Supplement: Supplementary file 1 [file genes-10-00696-s001.pdf]

Table 1. Primers for qRT-PCR analysis.

ID	Gene	Left Primer	Right Primer	Tm	GC%	Product size(bp)
TraesCS3A02G254600	TaDehydrin-1	TACGCTTATGAGGGCATGGTCGGCAGTG	TTCTTCCTCTGCCTCCCGCCTTGC	60	45	164
TraesCS3B02G286600	TaDehydrin-3	GGCGACCAGATCCAGCCCACCAAAGA	TCTTCCTCTGCCTCCCGCCTTGCCCGTCATCCTC	60	61	124
TraesCS3D02G255500	TaDehydrin-5	CGGCGACCAGATCCAGCCCACCAAA	TTCTTCCTCTGCCTCCCGCCCTGCCCGTCAT	68	45	126
TraesCS4A02G250900	TaDehydrin-7	TCGGCATCCAGTTGACAACTCAATT	GCCATCGCTTCACGGACTGCTTGCTG	60	55	90
TraesCS2A02G449700	TaLEA1-1	CGCGGCGACCACGCACGGCGAAAAG	AGATGTACTTCTCCGCCGCCGGGTG	60	45	233
TraesCS2B02G471500	TaLEA1-3	CGGCCGAGGTGCAGGGCAAGGCGGG	CGGGTACGCGGGGTCAGGGGCCACA	60	36	277
TraesCS4A02G129100	TaLEA4-13	CACCGCAAGGGCAAGATGTGAGCG	GACGGCATGGATGGCACGAGAACG	59	45	108
TraesCS1B02G237400	TaLEA5-1	AGCATCAGGCAATCACAAAGCAACA	GTCGGCGAGGTGCTCCTGCGCCTCG	60	45	215
TraesCS1D02G225800	TaLEA5-3	CACAGAACACCAACAGCAGTCGCATACC	TCTCCCCCTCGCGGGCCATGCGGTC	56	43	149
TraesCS3B02G166400	TaLEA5-4	GGAGGGCGAGACCGTCATCAAGAGC	CTCGAGCTTCTTCTCGTCCGGCTCG	62	60	172
TraesCS4A02G030600	TaSMP-1	ACGTGCTCTCGAATGCAACGGCGAA	GCCTCACCGTCTTGTCGTCCTTGTTTCG	63	55	114
TraesCS4A02G030700	TaSMP-2	ATGTGCCCTTCGTGATTGCTGATG	AATGTAGATGATGCCACGCTCTGCA	60	58	140
Ta54225	TaACTIN1	GTTCTACAACGAGCTCCGTGTC	GACATACATTGCTGGGCAAC	56	55	185

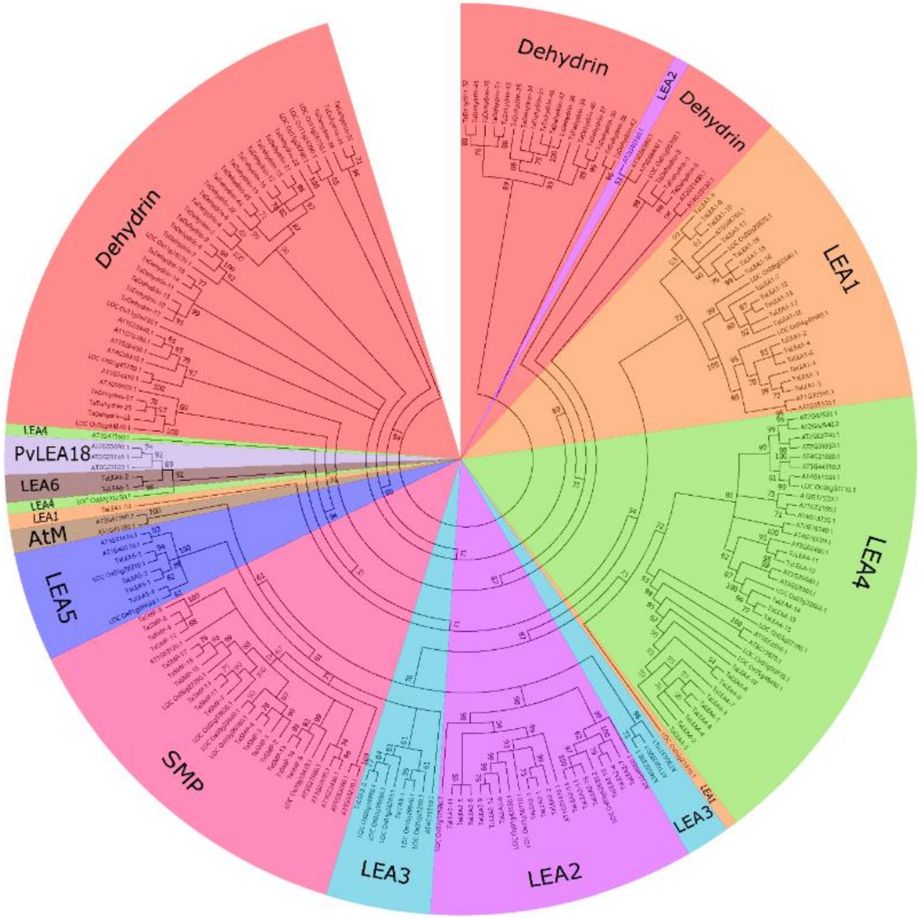

Figure S1. Phylogenetic analysis of LEA proteins among wheat (121), Arabidopsis (51) and rice (34) (by ML method).

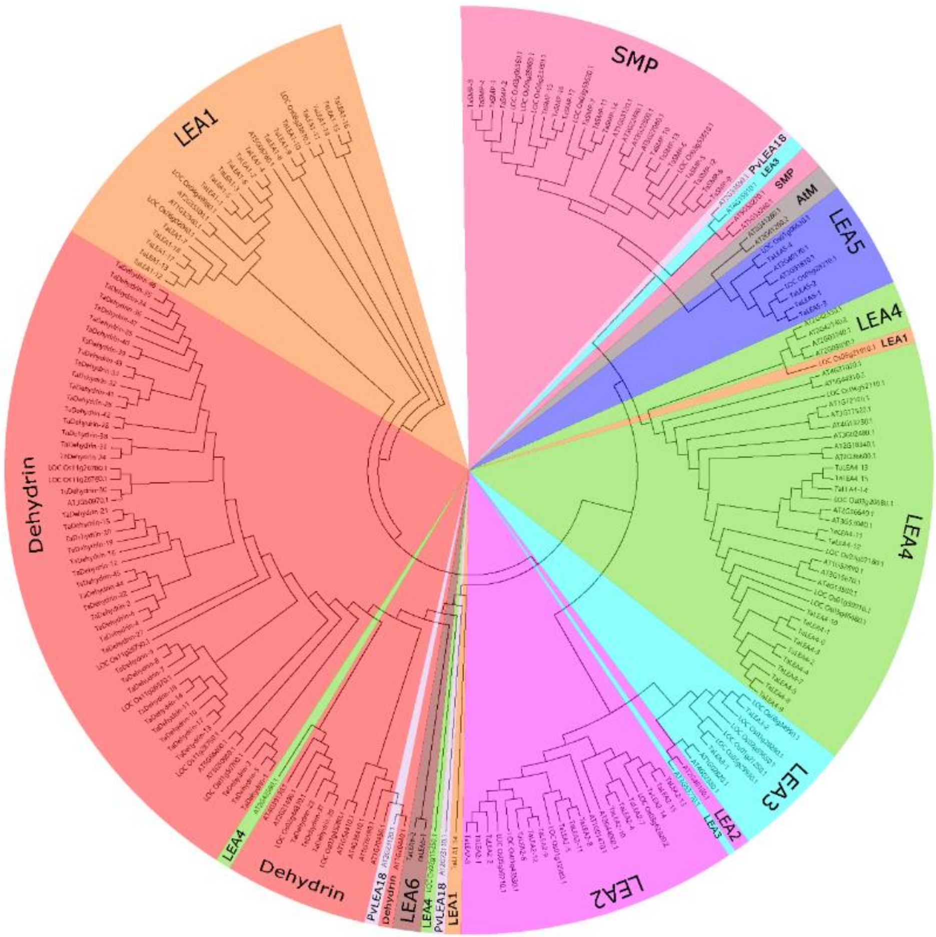

Figure S2. Phylogenetic analysis of LEA proteins among wheat (121), Arabidopsis (51) and rice (34) (by MP method).
